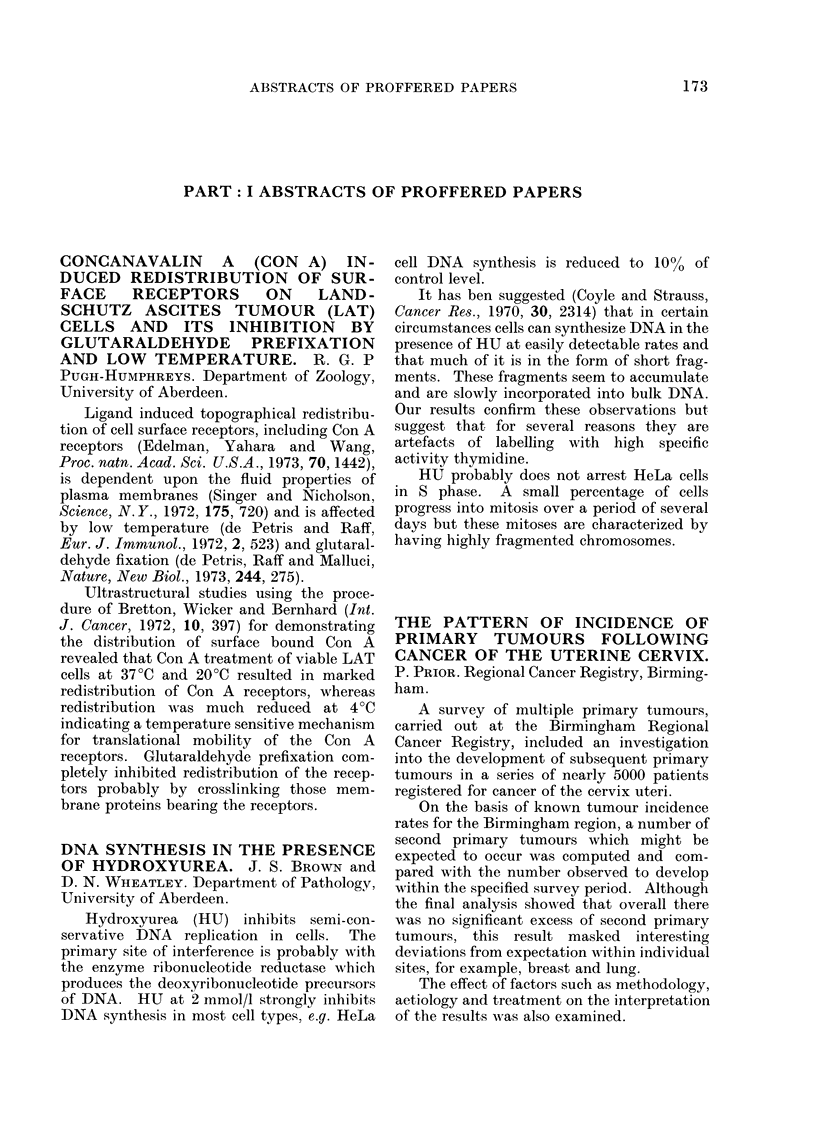# Proceedings: DNA synthesis in the presence of hydroxyurea.

**DOI:** 10.1038/bjc.1974.133

**Published:** 1974-08

**Authors:** J. S. Brown, D. N. Wheatley


**DNA SYNTHESIS IN THE PRESENCE OF HYDROXYUREA.** J. S. BROWN and D. N. WHEATLEY. Department of Pathology, University of Aberdeen.

Hydroxyurea (HU) inhibits semi-conservative DNA replication in cells. The primary site of interference is probably with the enzyme ribonucleotide reductase which produces the deoxyribonucleotide precursors of DNA. HU at 2 mmol/l strongly inhibits DNA synthesis in most cell types, *e.g.* HeLa cell DNA synthesis is reduced to 10% of control level.

It has ben suggested (Coyle and Strauss, *Cancer Res.*, 1970, **30**, 2314) that in certain circumstances cells can synthesize DNA in the presence of HU at easily detectable rates and that much of it is in the form of short fragments. These fragments seem to accumulate and are slowly incorporated into bulk DNA. Our results confirm these observations but suggest that for several reasons they are artefacts of labelling with high specific activity thymidine.

HU probably does not arrest HeLa cells in S phase. A small percentage of cells progress into mitosis over a period of several days but these mitoses are characterized by having highly fragmented chromosomes.